# Medical Items

**Published:** 1896-03

**Authors:** 


### MEDICAL ITEMS.

WE regret to chronicle the illness of our friend Dr. J. N. Tanlunson, and hope he will soon be restored to health.

DR. JAMES N. ELLIS, of Richmond, and Miss Leila Venable, of Atlanta, were married in Atlanta February 12th.

WE direct your attention to the special course at the Chicago Polyclinic and Hospital. For particulars see their advertisement.

THE *Clinical Recorder* is the name of a new quarterly journal to be published in New York, and edited by Dr. W. S. Gottheil. The first number contains some good articles, but the mechanical execution is miserable.

THERE will be a meeting of the alumni of the Atlanta Medical College, in the college building, at 12 o'clock March 31, for organization of a permanent Alumni Association. All alumni of the college are urged to be present.

THE papers announce the death of a lady in Chattanooga, February 18, from chloroform inhalation. She was about to be operated upon for some growth on the jaw, but died in few minutes after the anesthetic was begun. "Heart failure" is assigned as the cause of death.

CONGRESS has been requested to make an appropriation for a revised edition of the *Medical and Surgical History of the War of the Rebellion.* This elegant work has long been out of print. Write to your Congressman and ask his influence for this work. There should be another edition.

THE Apollinaris Company have kindly sent us some samples of the new Hunyadi water, which they are introducing under the name of *Apenta*. The chemical formula shows it to be principally a strong solution of sulphate of soda and sulphate of magnesia, the latter predominating.

---

THERE is a movement on foot to form a "National Confederation of State Examining and Licensing Boards," with the purpose "of reciprocal interstate action on the part of the State examining boards." Dr. Charles McIntire, of Easton, Pa., is chairman of the committee, and invites correspondence.

---

P. BLAKISTON, SON & Co., of Philadelphia, announce a book on "Appendicitis," by John B. Deaver, M.D., Assistant Professor of Applied Anatomy, University of Pennsylvania; Assistant Surgeon to the German Hospital, etc. The book will be arranged in a practical and systematic manner. The History, Etiology, Symptoms, Diagnosis, Operative Treatment, Prognosis, and Complications of this disease will be given in the order named. It will contain about forty illustrations of methods of procedure in operating, and typical pathological conditions of the appendix, the latter being printed in colors.

---

WE said in a late issue that we intended to publish the names of druggists known to be guilty of substitution. We have been furnished with the following:

Stegner Drug Co., cor. Grand and Easton avenues, St. Louis.
J. W. Crain, 75 West Madison avenue, Chicago.
International Pharmacy, 422 South Clark street, Chicago.
Merz Bros., southwest cor. Twelfth st. and Ogden ave., Chicago.
Richard Jentzsch, Twelfth and Ogden ave., Chicago.
Stolz & Grady, 104 North Clark ave., Chicago.
Charles H. Schad, 344 East Washington street, Indianapolis.
Charles S. Hayer, Fifth ave. and York street, Louisville.
E. Kampfmueller, Seventh and Broadway, Louisville.

MEDICAL boards should look out for diplomas and graduates of the "Illinois Health University." The business of this so-called university is chiefly to sell medical degrees without any sort of examination or qualification. The institution is located in Chicago, and it is said to advertise especially in the Detroit papers.

---

WE regret to learn of the death of Professor Joseph Jones, of New Orleans, which occurred February 19. Dr. Jones was born in Liberty county, Georgia, in 1833. In 1853 he graduated with distinction at Princeton College, and two years latter received his medical diploma from the medical department of the University of Pennsylvania. In 1856 he became Professor of Chemistry in the Savannah Medical College, serving two years, when he was elected Professor of Chemistry and Geology in the State University at Athens. The next year he was called to the chair of chemistry in the Medical College of Georgia at Augusta, which position he filled with great credit until the war. After one year as surgeon to several cavalry organizations in Georgia he was commissioned full surgeon in the Confederate army in 1862. He moved to New Orleans in 1868, and become Professor of Chemistry in the Universty of Louisiana, now Tulane University, and he has continued in this position since that time. Dr. Jones was a tireless worker and a voluminous writer on medical and scientific subjects. In 1876 Dr. Jones began the publication of his "Medical and Surgical Memoirs," containing valuable papers relating to the causes, nature, and management of various diseases, especially epidemic and contagious diseases, and a full exposition of the quarantine and sanitary laws necessary for the control of these diseases. In late years Dr. Jones has been President of the Louisiana Board of Health, and during his term of office the quarantine and sanitary regulations which he instituted succeeded in excluding yellow fever from the region of the lower Mississippi. Dr. Jones was one of the most learned men in our profession, and his reputation and labors were known on both continents. He died in his sixty-third year, esteemed and honored by all his contemporaries.

---

A Georgia Medical Poet.—We are indebted to Mr. Joel Benton (*Collier's Weekly*, October 31 and November 7, 1895) for some interesting information concerning Dr. Thomas Holley Chivers, a Georgia physician and poet, who lived, practiced medicine, and wrote poems in this State about sixty years ago, and whom the writer styled the "Precursor of Poe." More than this, however, little seems to be now known of Dr. Chivers. Many of his poems show the same effect of rhyme and meter that characterized the prettiest of Poe's poems about the same time or later. We give two stanzas from the "Vigil of Aiden," a poem covering twenty-six pages:

In the rosy bowers of Aiden,
With her ruby lips love-laden,
Dwelt the mild, the modest maiden
Whom Politian called Lenore.
* * * * *
For her lily-limbs so tender,
Like the moon in her own splendor
Seemed all earthly things to render
Bright as Eden was of yore.
* * * * *
Then he cried out, broken-hearted,
In this desert world deserted,
Though she had not yet departed,
"Are we not to meet, dear maiden,
In the rosy bowers of Aiden,
As we did in days of yore?"
And that modest, mild, sweet maiden,
In the rosy bowers of Aiden,
With her lily lips love-laden,
Answered, "Yes, forever more!"
And the old-time towers of Aiden
Echoed, "Yes, forever more!"

